# Exploring knowledge of first aid in epistaxis—25 years on

**DOI:** 10.1371/journal.pone.0315092

**Published:** 2025-01-15

**Authors:** Henry Dunne, Michael Abouabdallah, Joseph Roscamp, Samuel Birks, Kate Mcgibbon, Sam Dewhurst, David Strachan, Rishi Sharma

**Affiliations:** 1 Department of Clinical Neurosciences, Cambridge University, Cambridge, United Kingdom; 2 Department of Otolaryngology, Cambridge University Hospitals Trust, Cambridge, United Kingdom; 3 Department of Otolaryngology, Hull University Teaching Hospitals NHS Foundation Trust, Hull, United Kingdom; 4 Emergency Department, Sheffield Teaching Hospitals NHS Trust, Sheffield, United Kingdom; 5 Emergency Department, Mid and South Essex NHS Trust, Chelmsford, United Kingdom; 6 Otolaryngology Department, Northwest Anglia NHS Trust, Peterborough, United Kingdom; 7 Department of Otolaryngology, Bradford Royal Infirmary, Bradford, United Kingdom; Taichung Veterans General Hospital, TAIWAN

## Abstract

**Background:**

Epistaxis is the most common acute disorder managed by ENT services. A 1998 survey (Strachan and England) demonstrated widespread ignorance of correct first aid amongst the public with only 11% of respondents applying correct first aid techniques. Here we repeated and expanded the 1998 study to investigate whether understanding of correct first aid in epistaxis amongst the public and emergency department staff has improved in the last 25 years.

**Methods:**

Posters with links to surveys were displayed in ED waiting rooms, anticoagulation clinics and ED staff rooms in multiple UK centres. Responders were asked three first aid questions: pinch position, head position, and plugging nostrils. A prospective audit was carried out in a single centre over four weeks recording the first aid was being applied at the point of ENT review for patients referred with epistaxis.

**Findings:**

129 members of public responded. 83% do not have correct first aid technique including 77% of those on anticoagulants or aspirin. 116 ED staff responded. 64% do not use correct first aid. Over four weeks 19 patients were referred to ENT with epistaxis and of these, nine were bleeding at the point of ENT review. Adequate first aid was not being applied in 56% of those cases.

**Conclusions:**

Despite the morbidity of epistaxis, and the simplicity of first aid steps, there is concerning lack of understanding amongst the public and ED staff. Education (particularly for staff and the anticoagulated) may reduce emergency attendance in epistaxis patients.

## Introduction

The most common ENT presentation to emergency services in the UK is epistaxis [1] and it is associated with significant morbidity [2]. First aid measures as recommended by the National Institute for Health and Care Excellence (NICE) [3], Patient UK [4] and the Royal College of Emergency Medicine (RCEM) [5] include the same two simple steps. Applying pressure to the alar cartilage of the nose just above the nostrils and leaning forwards. Evidence in the form of controlled trials behind the efficacy of these recommendations is lacking [6]; nevertheless, given their logic, ease and low risks of application, this first aid method is supported by expert panel opinion in the British Rhinological Society (BRS) [1].

In 1998, Strachan and England evidenced the fact that understanding of these methods in the public was poor. At this time only 11% of those surveyed answered correctly that they would lean forwards and apply pressure to the alar cartilage in the event of epistaxis [7].

In 1993, McGarry and Moulton found that only 43% of trained emergency department staff in a single UK centre had correct understanding of first aid steps in epistaxis [8]. More recently, a single-centre study in the UK found that most emergency department staff still do not apply correct first aid methods [9].

The information landscape has change dramatically in the last two decades. Dissemination of information has changed from printed text and television to internet-based resources on handheld personal devices. 25 years on from Strachan and England’s initial assessment of the public’s understanding of first aid in epistaxis, the primary aim of our study was to repeat their study to identify if this knowledge gap remains as widespread in the internet age. Secondary aims were to evaluate the level of understanding of first aid for epistaxis amongst patients taking aspirin or anticoagulant medications and ED staff. Focus was made on these groups, due to the increased likelihood of epistaxis and hospital admission in the first group [10, 11] and the critical role in caring for epistaxis patients of the second group. This was done across multiple centres in the UK to expand upon previous single centre studies’ findings that ED staff understanding of first aid in epistaxis can be significantly improved upon. Finally, we completed a prospective audit of first aid being applied to patients with epistaxis after referral to the Ear Nose and Throat (ENT) team in a tertiary centre to explore how this understanding of first aid is being applied in practice.

## Methods

### Ethics statement

This project was given favourable ethical opinion by the East of England Cambridge South research and ethics committee. Consent was taking through an online link for the questionnaire which was the limit of the participants involvement. Participants confirmed they were over 16 years in order to participate and the need for further parental consent for those under 18 was not required as per the ethics committee. This consent process was approved by the East of England Cambridge South research and ethics committee.

### Survey study

From 1^st^ March 2022 till the 30^th^ June 2022, posters for healthcare users with QR code links to online Qualtrics© questionnaires were displayed in emergency department waiting rooms and anticoagulation clinics in multiple sites across the UK. Questionnaires were based on those which had previously been devised by Strachan and England. A poster for ED staff with a link to an adjusted questionnaire was displayed in ED staff rooms over the same time-period. Upon entering the questionnaire, participants were provided information about the study and asked to confirm that they were 16 years old or over and agree to take part in this study. If they indicated they consented to take part in the study this response was recorded and they were then able to complete the questionnaire.

Qualtrics© survey data was analysed in Excel and Fisher’s Exact test used during statistical analysis.

### Prospective audit

Over 4 weeks in April 2022, ENT junior trainees at Cambridge University Hospital NHS trust recorded details of first aid being applied at the point of their review for any patient referred to ENT with epistaxis. They were also asked if this was adequate or inadequate based on RCEM and NICE guidance for first aid in epistaxis.

## Results

### Healthcare users

129 healthcare users responded to the questionnaire from six sites (Basildon 1, Bradford 6, Cambridge 64, Chelmsford 46, Peterborough 2, Sheffield 10).

**Fig 1:**
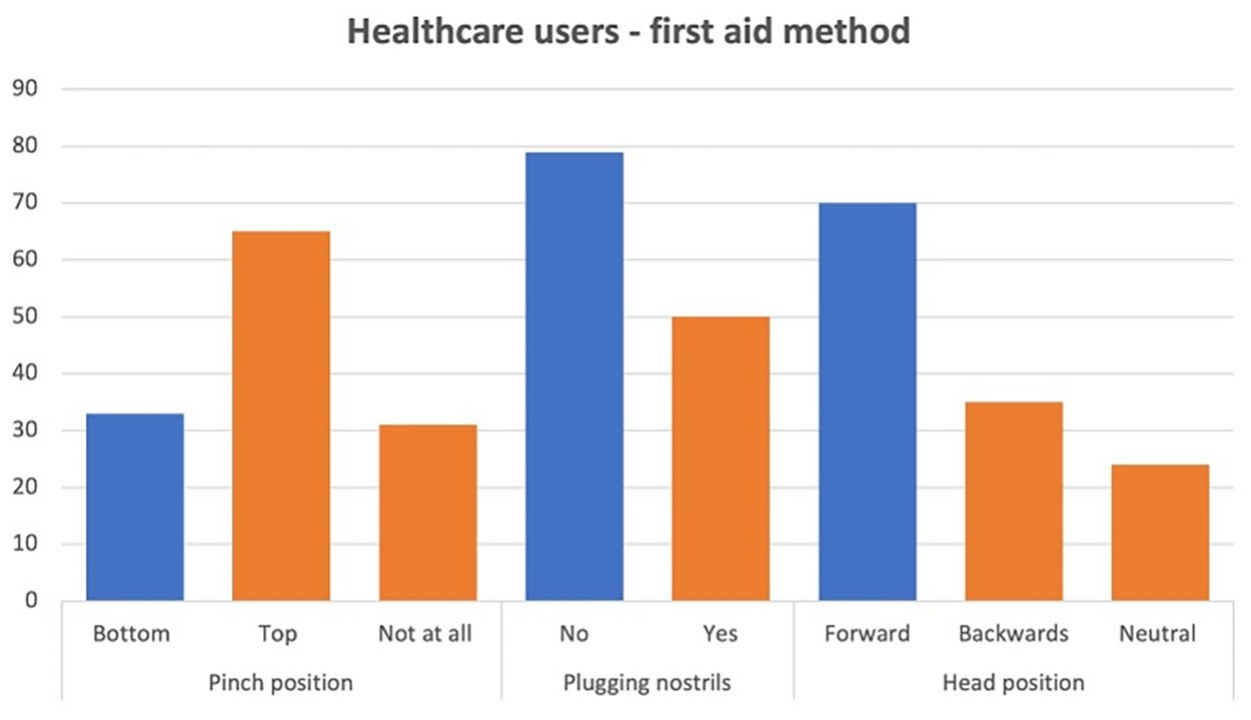
Healthcare users questionnaire responses. First aid methods.

**Fig 2:**
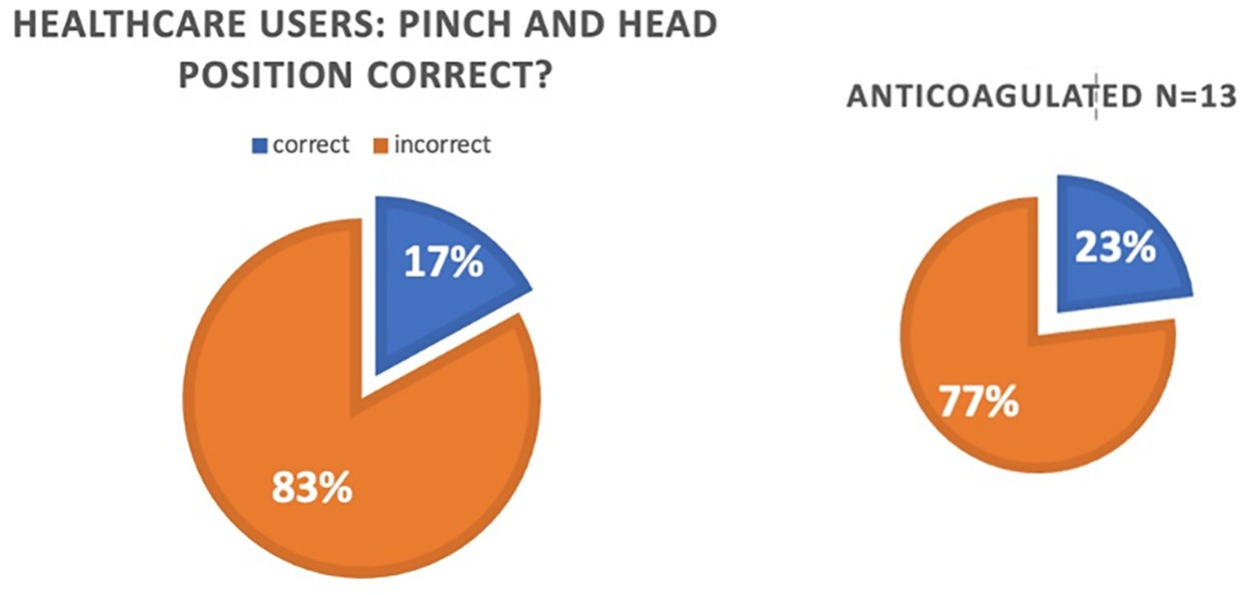
Proportion of healthcare users, and healthcare users who take anticoagulants or aspirin with correct and incorrect first aid methods.

Fig 1 outlines responses to questions regarding how they would go about arresting a nosebleed. Fig 2 shows the overall proportion of responders who had the correct response (holding head forwards and pinching lower down).

In comparison to the 1998 study by Strachan and England, there was a statistically significant improvement, although slight, in proportion of the healthcare users who use correct first aid technique: 17% vs 11%. (p = 0.044).

**Fig 3:**
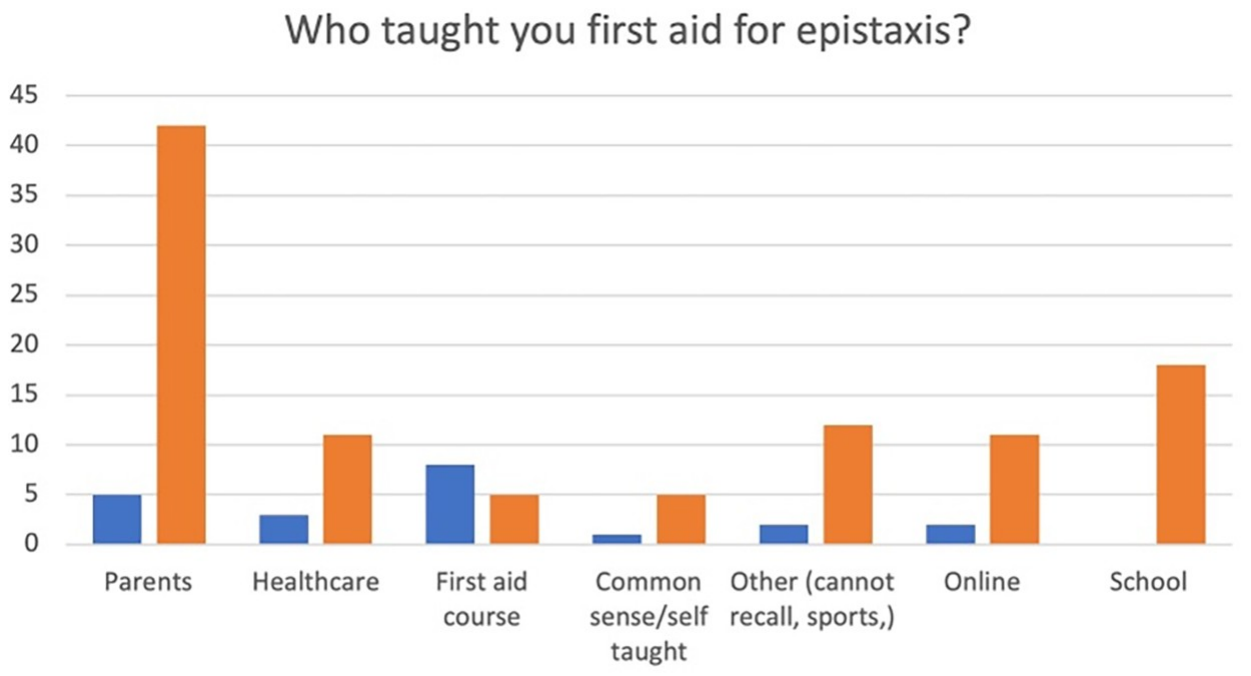
Healthcare users’ responses to the question: ‘Who taught you first aid for epistaxis?’.

Fig 3 shows from whom healthcare users learnt first aid and highlights that 11/14 (79%) of responders who report they had been taught by other healthcare staff apply incorrect first aid.

### Anticoagulation patients

13 of the 129 healthcare users indicated that they took regular anticoagulation medication, or aspirin (Warfarin 4, Apixaban 1, Aspirin 8). Of these, only 3 (23%) use the correct method of first aid to arrest nosebleeds (Fig 2). There was no statistically significant difference in rate of correct understanding of first aid for epistaxis in responders who take anticoagulation and those who do not (P = 0.16).

### Emergency department staff

116 emergency department staff from multiple centres responded to the survey. (Addenbrooke’s, Cambridge 49; Bradford Royal Infirmary 12; Broomfield General Hospital, Chelmsford 15; and Northern General, Sheffield 40). This included 27 Doctors, 71 nursing staff members and 7 paramedics. 107 (92%) responders indicated that they had been involved in the management of a patient with epistaxis.

**Fig 4:**
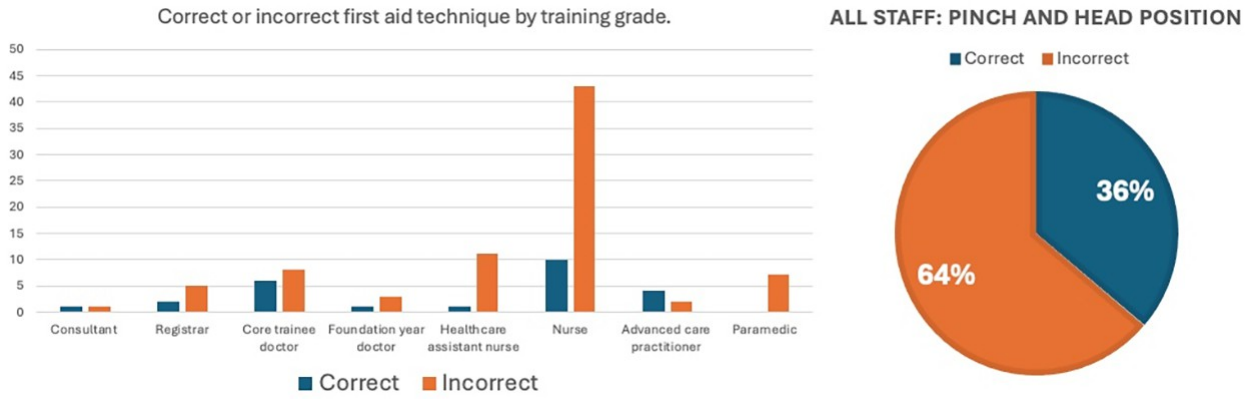
Proportion of emergency department staff with correct and incorrect first aid technique in epistaxis.

Fig 4 outlines responses to the question regarding applying first aid to a patient experiencing epistaxis. Fig 4 outlines the overall proportions of staff member groups with incorrect first aid technique including 63% of ED doctors and 79% of ED nurses. There was no statistically significant difference between doctors and nurses in terms of knowledge of correct first aid technique (p = 0.89).

### Prospective audit of first aid

Over the 4-week data collection period, 19 patients were referred to the ENT team with epistaxis. Of these, nine were actively bleeding at the point at which the ENT speciality trainee assessed the patient. Of those actively bleeding, ENT speciality trainees reported five (55%) were not having adequate first aid applied.

## Discussion

Correct first aid for epistaxis is straightforward and simple to apply. Described simply, pressing the soft part of the nose applies pressure and tamponades bleeding points over Little’s area (the vascular plexus at the anterior nasal septum), which is the source of bleeding in 90% of cases of epistaxis [12, 13]. Leaning forwards reduces the volume of blood that might be being inhaled or swallowed and allows a patient to spit out blood that may enter the oropharynx via the nasopharynx. Evidence has clearly shown that correct first aid should stop the majority of nosebleeds [7], and therefore prevent the need to present to hospital. It therefore follows that applying this evidence can reduce demand on already overburdened Emergency Departments. After identifying a large knowledge gap amongst the public in 1998, Strachan and England called for better education of the public and training for healthcare workers to improve understanding of correct first aid for epistaxis.

Our study was limited by the number of responses and therefore some of the subgroup analysis have relatively small numbers. Nevertheless, we demonstrate that throughout the UK, knowledge of correct first in epistaxis amongst the public and healthcare users remains an area for significant improvement with only 17% of those responding having correct first aid technique. We find that those on aspirin or anticoagulant medications, with a high risk of epistaxis [10, 11], are no better informed than the other healthcare users. We propose that prior to starting these therapies, patients should receive written and practical demonstrations of epistaxis first aid measures. This simple educational intervention would not add significant time to anticoagulation consultations but is likely to have a significant impact upon emergency healthcare utilisation in this patient group.

Perhaps of more concern we find only 36% of emergency department staff across multiple centres apply correct first aid when managing patients with epistaxis. These findings are comparable to previous single-centre studies, each of which finds less than 45% of emergency department staff have correct first aid technique [8, 9]. Our multi-site study shows that this is a consistent finding across multiple geographical areas. This suggests a lack of emphasis on epistaxis first aid in medical and nursing curricula given that the first aid techniques are widely disseminated and easily available. More concerningly, our prospective audit found that in more than 50% of patients experiencing active epistaxis, first aid was being applied incorrectly. This provides direct evidence of how this knowledge gap directly impacts patients. In a health service currently experiencing unprecedented demands, simple first aid measures could free up resources and allow more efficient utilisation of healthcare workers.

Another concern regarding the lack of understanding amongst healthcare staff, is related to their role in clinical education and public health. Dissemination of incorrect advice on first aid in epistaxis acts as a ’force-multiplier’ compounding the problem. This is demonstrated in our data, with 79% of those who reported learning how to manage epistaxis from healthcare staff in fact doing so incorrectly.

The fact that high levels of ignorance of correct first aid in epistaxis has persisted in both healthcare users and workers over the past 25 years is concerning. In an era where almost all the world’s knowledge is instantaneously available to people through smart devices, accessing information should no longer be an issue. Even where smart phone penetrance is lower, for example, in older or more deprived groups, there is plenty of scope to target appropriate first aid advice where it is needed. First aid demonstrations would add little additional time to those at risk. Targeted ENT specialist delivered training to anticoagulation clinics, first aid groups (e.g., St John’s Ambulance teams) would be an efficient way to achieve this. We support Jamshaid et al’s recommendation for a need for enhanced education at grassroots levels [9] and reiterate Strachan and England’s recommendation to improve education for healthcare users and workers alike [7].

Accurate first aid is vital in minimising the morbidity of epistaxis. The knowledge gap amongst both patients and healthcare staff remains considerable. The fact that this remains the case 25 years on after first being identified is disappointing. That this is the case in an era where information is easily available and accessible makes this even more unsatisfactory. An increased emphasis on emergency and primary care curricula is essential to ensure we improve standards in the next generation.

## Supporting information

S1 File Data file. (XLSX)
